# On the Need for Deconvolution Analysis of Experimental and Simulated Thermoluminescence Glow Curves

**DOI:** 10.3390/ma16020871

**Published:** 2023-01-16

**Authors:** George Kitis, Vasilis Pagonis

**Affiliations:** 1 Nuclear Physics and Elementary Particles Physics Section, Physics Department, Aristotle University of Thessaloniki, 54124 Thessaloniki, Greece; 2 Physics Department, McDaniel College, Westminster, MD 21157, USA

**Keywords:** thermoluminescence, stimulated luminescence, kinetic parameters, superposition principle, competition between levels, computerized glow curve deconvolution

## Abstract

Simulation studies of thermoluminescence (TL) and other stimulated luminescence phenomena are a rapidly growing area of research. The presence of competition effects between luminescence pathways leads to the complex nature of luminescence signals, and therefore, it is necessary to investigate and validate the various methods of signal analysis by using simulations. The present study shows that in simulations of luminescence signals originating from multilevel phenomenological models, it is not possible to extract mathematically the individual information for each peak in the signal. It is further shown that computerized curve deconvolution analysis is the only reliable tool for extracting the various kinetic parameters. Simulation studies aim to explain experimental results, and therefore, it is necessary to validate simulation results by comparing with experiments. In this paper, testing of simulation results is performed using two methods. In the first method, the influence of competition effects is tested by comparing the input model parameters with the output values from the deconvolution analysis. In the second method, the agreement with experimental results is tested using the properties of well-known glow peaks with very high repeatability among TL laboratories, such as the 110 °C glow peak of quartz.

## 1. Introduction

Simulation studies of thermoluminescence (TL) and other stimulated luminescence phenomena are a rapidly growing area of research [1,2]. Simulations of phenomenological models consisting of many energy levels responsible for TL peaks result in complex TL glow curves, which are very similar to experimental glow curves. In both experimental and simulated glow curves, it is important to extract the information regarding the TL intensity used for dosimetric applications, as well as the kinetic parameters of each peak, which can then be used to evaluate the signal lifetimes for specific dosimetric applications [3].

**Figure 1:**
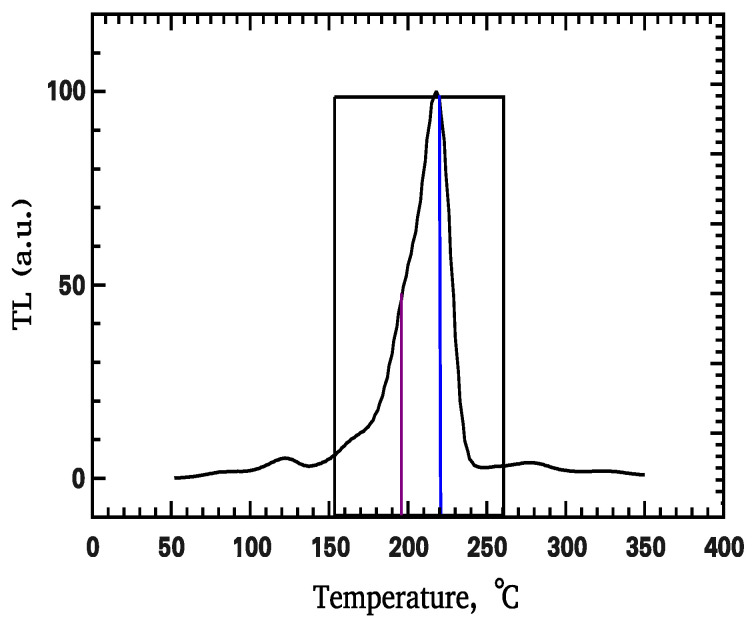
Traditional methods to extract the TL intensity from experimental glow curves either by selecting the peak height at some point of the glow curves (perpendicular lines) or by integrating the signal between two temperatures (region within the box).

In traditional TL literature, the TL intensity from experimental glow curves is evaluated (Figure 1) by selecting the intensity at some point of the glow curves (termed the peak height), or by integrating the signal between two temperatures [3,4].

During the last decade, the technique of computerized glow curve deconvolution (CGCD) has been applied extensively [5,6,7,8], although some textbooks have raised objections to its wide application [9,10,11]. The skepticism shown by some researchers is based on the fulfillment or not of the superposition principle (SP), which postulates that the energy levels responsible for each individual TL peak do not depend on each other.

In this work, the best techniques for analyzing the complex glow curves resulting from a simulation will be investigated, attempting to answer the question: is it possible to analyze simulated TL signals using the same methods as for experimental TL signals?

The aims of the present work are: (i) To simulate complex TL glow curves and investigate whether it is possible to extract the quantitative characteristics of each component in the TL signal; (ii) To study the influence of competition effects between traps on the parameters extracted from the simulated TL signals; (iii) To investigate the relation between the input kinetic parameters in the model, and the output values obtained by analyzing the TL signals using CGCD methods; (iv) To establish criteria for the validity of the results obtained in TL simulations; (v) To examine how experimental TL glow curves widely available in the literature can be used for testing the results from simulations.

## 2. Materials and Methods

### 2.1. The Phenomenological Model

**Figure 2:**
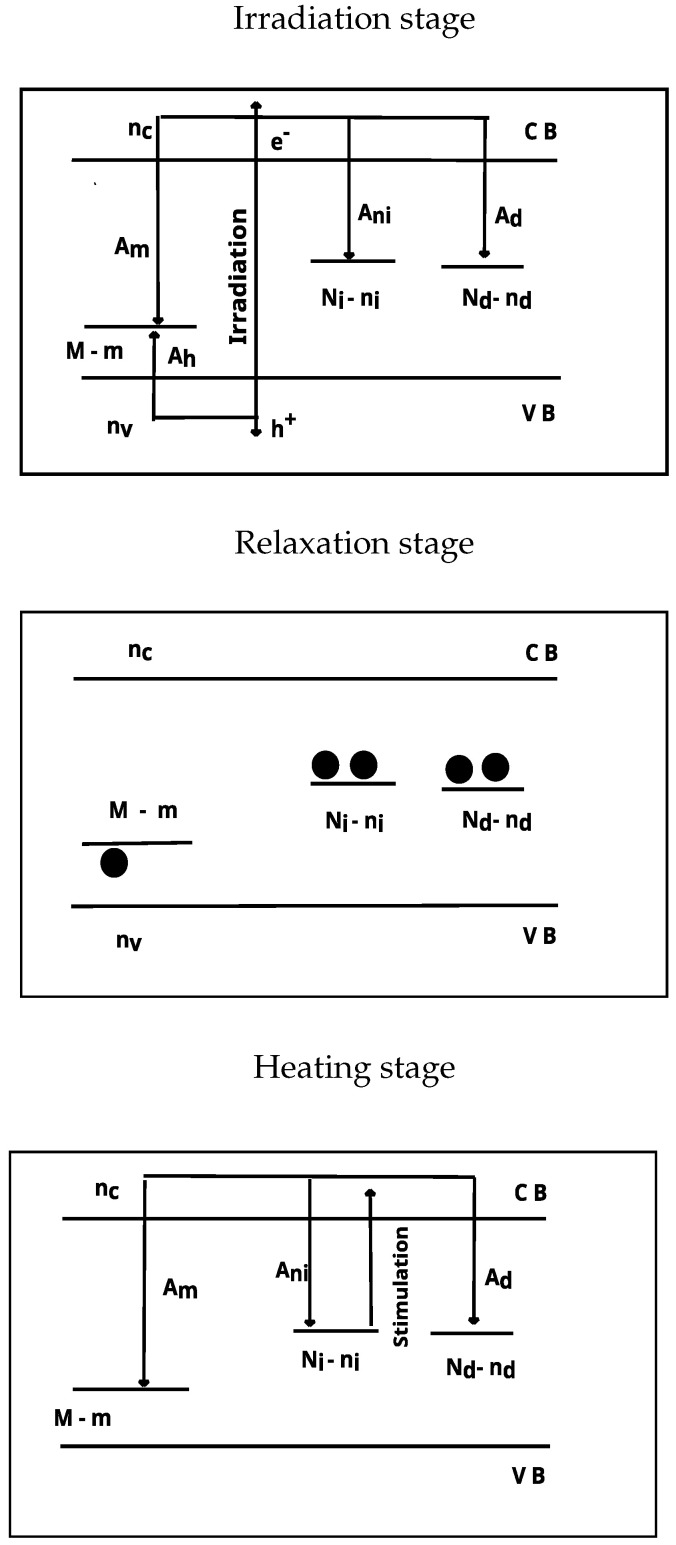
Energy band model used for simulation. (**Upper**): Irradiation stage. (**Middle**): Relaxation stage. (**Down**): Heating stage.

The simulation model used in this paper consists of i=1,⋯,6 electron traps and one hole trap [3]. The electron traps which can both trap and release electrons are termed active traps, whereas electron traps which can trap only electrons without releasing them at the temperature region of the active traps are termed thermally disconnected deep traps (TDDT). The hole trap in the model acts also as the recombination center. Figure 2 show schematically the energy levels for this model. The simulation consist of three stages termed the irradiation stage, relaxation stage and heating stage. The differential equations governing the traffic of electrons and holes in this model are:

(1) $$\begin{array}{ccc} \frac{dT}{dt} & = & \beta \end{array}$$

(2) $$\begin{array}{ccc} \frac{dR}{dt} & = & Drate \end{array}$$

(3) $$\begin{array}{ccc} \frac{dn_{i}}{dt} & = & -\sum n_{i}\;s_{i}\;e^{-\frac{E}{kT}}+A_{i}\;\left(N_{i}-n_{i}\right)\;n_{c} \end{array}$$

(4) $$\begin{array}{ccc} \frac{dn_{d}}{dt} & = & A_{d}\left(N_{d}-n_{d}\right)\;n_{c}, \end{array}$$

(5) $$\begin{array}{ccc} \frac{dm}{dt} & = & A_{h}\;\left(M-m\right)\;n_{v}-A_{m}\;m\;n_{c}, \end{array}$$

(6) $$\begin{array}{ccc} \frac{dn_{v}}{dt} & = & Drate-A_{h}\;\left(M-m\right)\;n_{v}, \end{array}$$

(7) $$\begin{array}{ccc} \frac{dn_{c}}{dt} & = & Drate-\sum \frac{dn_{i}}{dt}-\frac{dn_{d}}{dt}-A_{m}\;m\;n_{c}, \end{array}$$

where the index i=1,⋯,5 stands for the active electron traps, $E_{i}\;\left(eV\right)$ is the activation energy, $s_{i}\;\left(s^{-1}\right)$ the frequency factor, $N_{i}\;\left({\mathrm{cm}}^{-3}\right)$ is the concentration of available electron traps, $n_{i}\;\left({\mathrm{cm}}^{-3}\right)$ the concentration of trapped electrons, $M\;({\mathrm{cm}}^{-3})$ is the concentration of available luminescence centers, $m_{i}\;\left({\mathrm{cm}}^{-3}\right)$ concentration of trapped holes. $N_{d},\;n_{d}\;\left({\mathrm{cm}}^{-3}\right)$ are the concentrations of available and occupied traps in a thermally disconnected deep trap (TDDT), $n_{c}\;\left({\mathrm{cm}}^{-3}\right)$ and $n_{v}\;\left({\mathrm{cm}}^{-3}\right)$ are the concentration of electrons in the conduction and holes in the valence band, $A_{i}\;\left({\mathrm{cm}}^{3}\;s^{-1}\right)$ are the trapping coefficients in electron traps $n_{i}$, $A_{m}\;\left({\mathrm{cm}}^{3}\;s^{-1}\right)$ is the recombination coefficient, $A_{h}\;\left({\mathrm{cm}}^{3}\;s^{-1}\right)$ is the trapping coefficient for holes in luminescence centers, $A_{d}\;\left({\mathrm{cm}}^{3}\;s^{-1}\right)$ trapping coefficient in TDDT, β(K/s) is the heating rate and Drate is the rate of production of ion pairs (i.p) per second (i.p/s) which is proportional to the dose rate.

Equation (1) evaluates the temperature as a function of time, whereas Equation (2) evaluates the generated (i.p/s) used in Equations (6) and (7). Equation (3) describes the trapping and thermal release of electrons by the active traps, while Equation (4) refers to the competitor TDDT.

In the above model, all traps with the index i=1⋯5 can trap and release electrons by thermal excitation, and they are referred to as active traps. On the other hand, the TDDT with index *d* can only trap electrons while thermal stimulation of electrons is not allowed.

In the present work, the TL signals are simulated by solving the system of ordinary differential equations (ODE) described by Equations (1)–(7). The system of ODE is solved using the standard Scientific Python (SciPy) package in the Python programming language.

We specifically simulate four reference TL glow peaks (REFERENCE-01 to REFERENCE-04), with the trap parameters listed below. The four reference glow curves were chosen so that the amount of competition between traps is highest for REFERENCE-01, and it decreases progressively up to REFERENCE-04:

- $N_{0}=\;2.5\times e^{10}$;
- $N_{1},\;N_{2},\;N_{3},\;N_{4},\;N_{5},\;N_{6}=0.1\times 10^{10},\;0.2\times 10^{10},\;0.2\times 10^{10},\;0.5\times 10^{10},\;0.5\times 10^{10},\;1.0\times 10^{10}$, in ${\mathrm{cm}}^{-3}$;
- $E_{1},\;E_{2},\;E_{3},\;E_{4},\;E_{5}=1.0,\;1.38,\;1.48,\;1.6,\;2.01$, in eV;
- $s_{1},\;s_{2},\;s_{3},\;s_{4},\;s_{5}=1\times e^{13},\;3.9\times 10^{16},\;2\times 10^{16},\;2\times 10^{16},\;4\times 10^{19}$ in $s^{-1}$;
- Drate = $2.5\times 10^{9}\;\;i.p/s$;
- REFERENCE-01-($A_{1,2,3,4}=10^{-9}$, $A_{5}=4\times 10^{-9}$, $A_{m}=A_{d}=10^{-7}$), in ${\mathrm{cm}}^{3}\;s^{-1}$;
- REFERENCE-02-($A_{1,2,3,4}=5\times 10^{-8}$, $A_{5}=9\times 10^{-8}$, $A_{m}=A_{d}=10^{-7}$), in ${\mathrm{cm}}^{3}\;s^{-1}$;
- REFERENCE-03-($A_{1,2,3,4}=10^{-9}$, $A_{5}=4\times 10^{-9}$, $A_{m}=10^{-7}$, $A_{d}=10^{-10}$), in ${\mathrm{cm}}^{3}\;s^{-1}$;
- REFERENCE-04-($A_{1,2,3,4}=5\times 10^{-8}$, $A_{5}=9\times 10^{-8}$, $A_{m}=10^{-7}$, $A_{d}=10^{-10}$), in ${\mathrm{cm}}^{3}\;s^{-1}$.

### 2.2. CGCD Analysis Method

The complex TL glow curves resulting from simulation, as well as a large number of experimental glow curves of natural quartz, were analyzed using the CGCD analysis technique. Specifically, the CGCD equation used here is based on the one trap one recombination center (OTOR) model. The solution of the OTOR model is based on Lambert W(Z) function [12,13] and was derived by Kitis and Vlachos [14]. Later, Singh and Gartia [15] developed a similar analytical equation based on the Wright function. The analytical equation was used in the following form, which was also previously used [3,4,16]:

(8) $$I=I_{m}\;\exp\left(\frac{E(T-T_{m})}{k\;T\;T_{m}}\right)\cdot \frac{W\left[e^{z_{1m}}\right]+W{\left[e^{z_{1m}}\right]}^{2}}{W\left[e^{z_{1}}\right]+W{\left[e^{z_{1}}\right]}^{2}}.\;$$

with

(9) $$z_{1}=\frac{R}{1-R}-ln\left(\frac{1-R}{R}\right)+\frac{E\;e^{\frac{E}{k\;T_{m}}}}{k\;T_{m}^{2}}\;\frac{F(T,E)}{1-1.05\;R^{1.26}}.$$

and

(10) $$F\left(T,E\right)=T\cdot exp\left(-E/kT\right)+\frac{E}{k}\cdot Ei\left(-E/kT\right)$$

where $T_{m}$ the temperature at the maximum peak intensity $I_{m}$, $R=A_{n}/A_{m}$ is the ratio or re-trapping over recombination coefficients and $z_{1m}$ is the value of $z_{1}$ at the peak maximum temperature $T_{m}$.

The function F(T,E) is the well-known integral appearing in TL theory, which is used here in terms of the built-in exponential integral function Ei(x), instead of the asymptotic series approximation used previously in the literature [1,9].

In modern software packages, the Lambert function W(z) is a built-in function, similar to any other transcendental function like sine, cosine, etc. The Lambert function is termed ProductLog[(0,1),z] in Mathematica, Lambert $w_{0}$ and $w_{1}$ in MATLAB and EXCEL, lambertw in Python, gsl-sf-lambert-$w_{0}\left(z\right)$, gsl-sf-lambert-$w_{1}\left(z\right)$ in GNU GSL. $w_{0}$ and $w_{1}$ stands for the first and second real branch of the Lambert function, respectively.

As z→∞, the numerical value of $e^{z}$ in the above equations overflows, and the Lambert function W(Z) in Python does not return a value. In such cases, the Lambert *W* can be approximated by

(11) $$W\left(e^{z}\right)=z-ln\left(z\right)$$

Most software packages have implemented the Wright ω(Z) function, and the above overflow issue is overcome by using the Wright ω(Z) function [15], instead of the Lambert function W(Z). The two functions are related according to:

(12) $$W\left(e^{z}\right)=\omega \left(z\right).$$

### 2.3. CGCD Analysis Software

The CGCD analysis was applied using the ROOT data Analysis Framework [17]. All TL glow curve fittings were performed using the MINUIT program [18] released in ROOT, which is a physics analysis tool for function minimization. The Lambert function W(z) and the exponential integral function $Ei[-\frac{E}{kT}]$ are implemented in ROOT through the GNU scientific library (GNU GSL) [19].

The goodness of fit was tested using the figure of merit (FOM) [20], which was initially proposed to test the goodness of fit of gamma ray spectroscopy data. Currently, the FOM is widely used by the TL /OSL community within the CGCD deconvolution analysis [21]. It is defined as

(13) $$FOM=100\%\times \frac{\sum \left|y_{i}^{0}-y_{i}^{f}\right|}{\sum y_{i}{}^{f}}$$

where $y_{i}^{0}$ corresponds to experimental points and $y_{i}^{f}$ to fitted points.

### 2.4. Materials and Experiments

Simulations and experiments must support each other. In the present work, we investigate whether experimental results can be used to validate simulation results. The original quartz samples were large crystals of hydrothermal and metamorphic origin which occur in vein—associated metamorphic rocks, collected from different locations spanning Africa (Nigeria), Europe (Greece) and Asia (Nepal) (more details in [22,23,24]). The experimental data analyzed here concern the low-temperature TL peak of quartz (the 110 °C TL peak), which was studied for both pre-dosed and natural aliquots.

All the TL measurements on the quartz samples were carried out using a RIS∅TL/OSL reader (model TL/OSL–DA–15) equipped with a 0.075 Gy/s${}^{90}Sr{/}^{90}Y$ beta ray source [25]. The reader was fitted with a 9635QA photomultiplier tube. The detection optics consisted of a 2.5 mm Hoya U–340 (kp 340 nm, FWHM 80 nm) filter.

The experimental protocol is as follows:

Step 1: Readout up to 250 °C at β = 1 °C/s.
Step 2: Irradiate with a small test dose (less than 1 Gy).
Step 3: Readout up to 250 °C at β = 1 °C/s.
Step 4: Give the same test dose of Step 2.
Step 5: Readout up to 500 °C at β = 1 °C/s.

## 3. The First Requirement for a Valid Simulation Test: Using Three Simulation Stages

Before presenting the results of the simulations, we first discuss the optimal method of carrying out TL simulations.

All TL simulations should contain three distinct simulation stages, namely, the irradiation stage, relaxation stage and the heating stage. The parameters in these stages should be set up as follows:

**Irradiation stage:**  Set the initial values of all parameters at time t = 0, i.e., $T_{0}$ = 273 K, $n_{i0}$ = 0, $m_{0}$ = 0, $nc_{0}$ = 0, $nv_{0}$ = 0. Set also β=0 since there is no heating, and set the irradiation dose rate (in the present work $Drate=10^{5}\;e\text{-}h\;\mathrm{pairs}/s$). Store the last values of all concentrations at the end of the irradiation stage, which will be used as the initial concentrations for the next stage.
**Relaxation stage:**  This stage simulates the time interval between the end of irradiation stage and the beginning of the heating stage. Set as initial values the last concentrations of the previous irradiation stage. Set also β=0 and Drate=0, since there is no heating and no irradiation. Store the last values of all concentrations at the end of this stage, which will be the initial concentrations for the next stage.
**Heating stage:**  Set as initial values the last values of the previous relaxation stage. Since there is no irradiation and hole trapping, set Drate=0 and $A_{h}=0$. Set the heating rate β (in our simulations β = 2 K/s). At the end of this stage, the TL glow peak is evaluated.

In order to show the importance of using three simulation stages, we will describe two cases in the literature, in which the three stage requirement is not followed.

The first example concerns the Randall–Wilkins model [26,27], which consists of only the heating stage. This model produces the very well known analytical equation for first-order kinetics. However, despite its great role in TL research, this model has a restricted physical basis. The reason is the requirement for zero re-trapping during the heating stage, which also means zero trapping during the irradiation stage. This means that the initial condition leading to the Randall–Wilkins model cannot be achieved at all during the irradiation stage. The Randall–Wilkins kinetics is an extreme boundary condition of the OTOR model.

The second example concerns TL simulation studies with extreme pessimistic results concerning the validity of the TL phenomenon itself [28,29,30]. For many decades, these pessimistic conclusions are, unfortunately, reproduced by several TL textbooks without any further study [9,10,11].

Sadek and Kitis [31] examined in detail the simulations of Kelly et al. [28,29] and Opanowitz [30] and they found that the common point of both studies was that they contain only the heating stage. Sadek and Kitis [31] investigated these previous results in two ways: first by deriving the TL peaks and then fitting them using analytical expressions for single TL peaks. Several of the TL peaks were fitted excellently, reproducing exactly the values of the activation energy used in the simulations, contrary to the conclusions of Kelly et al. [28,29] and Opanowitz [30]. On the other hand, there were cases of peaks which could be fitted well with the analytical equations but gave erroneous values of the activation energy, and also cases that was impossible to fit, in agreement with the pessimistic conclusions. In this last group of cases, Sadek and Kitis [31] rewrote the models of Kelly et al. [28,29] and of Opanowitz [30] so that they also contained the irradiation and relaxation stages. The final conclusion of Sadek and Kitis [31], after extensive trials, was that the parameters used by Kelly et al. [28,29] and Opanowitz [30] cannot be attained by using appropriate irradiation stages.

These two examples from the literature show that using three-stage simulations is a first necessary requirement for the validity of simulations. An arbitrary selection of parameter values for the heating stage should be avoided since it can easily lead to non-physical results.

## 4. Is It Possible to Evaluate the Contribution of Each Trap Using the Numerical Solution of the Differential Equations?

In this section, we discuss whether one can isolate the contribution of each trap to the TL signal mathematically by using the numerical solution of the differential equations. The simulated luminescence signal corresponds to the total number of recombination events in the luminescence centers, i.e.,

(14) $$TL\left(t\right)=A_{m}\;m\left(t\right)\;n_{c}\left(t\right)$$

In an attempt to separate the photons originating from each specific electron trap during the heating process, we use the neutrality condition and write Equation (14) in the form:

(15) $$TL\left(t\right)=A_{m}\;\left(n_{1}\left(t\right)+n_{2}\left(t\right)+n_{3}\left(t\right)+\ldots n_{i}\left(t\right)+n_{c}\left(t\right)\right)\;n_{c}\left(t\right)$$

Based on simple inspection of this equation, one may suppose that the contribution of each individual trap to the TL signal will be given by an expression of the form:

(16) $$TL_{1}\left(t\right)=A_{m}\;n_{1}\left(t\right)\;n_{c}\left(t\right);\;TL_{2}\left(t\right)=A_{m}\;n_{2}\left(t\right)\;n_{c}\left(t\right);\;TL_{3}\left(t\right)=A_{m}\;n_{3}\left(t\right)\;n_{c}\left(t\right);\;,etc.$$

However, this is incorrect, since the concentration of electrons $n_{i}$ from each trap is multiplied by $n_{c}\left(t\right)$, which is due to electrons originating not only from trap $n_{i}$, but from all traps. It is a basic property of the delocalized multilevel phenomenological models that once the electrons are released into the conduction band, they have no memory of their origin. As a result of this memory loss, the correspondence between the number of trapped electrons $n_{i}$ and the number of recombined electrons is lost.

The basic consequence of this situation is that it is not possible to extract mathematically from the numerical solution of the differential equations useful information regarding the intensity of the individual components in the TL signal. Due to the presence of the term $n_{c}$, the individual peaks in the TL signal are correlated to each other. This contradicts the superposition principle (SP), which is an underlying assumption during the CGCD analysis of complex TL signals. The validity of the SP requires careful examination, and it is discussed in the next section.

## 5. Is the Superposition Principle Valid When We Apply CGCD Methods of Analysis?

Unfortunately, the computerized techniques for analysis of TL glow-curves are not universally accepted. A part of the TL community argues that deconvolution is valid only when the superposition principle holds [9,10,11]. According to these authors, this happens only for the Randall–Wilkins type of first-order kinetics [26,27]. We wish now to discuss and clarify this point, before continuing with the two main objectives of the present work.

The SP states that at a given place and time, the response due to two or more stimuli equals to the sum of the response that would have been caused by each stimulus individually [32]. Mathematically, the SP is expressed as

(17) $$f\left(x_{1}+x_{2}+\cdots +x_{n}\right)=f\left(x_{1}\right)+f\left(x_{2}\right)+\cdots +f\left(x_{n}\right).$$

where *x* is a parameter and y=f(x) is the respective response.

As is obvious from Equations (1)–(7), the shape of complex TL curves is due to the competition among electron traps. The existence of competition makes them correlated, and therefore, the basic requirement of independence assumed by Equation (17) is lost. One would then be tempted to conclude that the SP does not hold and that the CGCD method of analysis cannot be applied.

Sadek and Kitis [32] used a phenomenological model similar to the model used in the present work, and simulated in detail the impact of the non-fulfillment of SP on the analysis of TL glow curves. The study of Sadek and Kitis [32] was based on varying the amount of competition between traps, which is controlled by the parameters $A_{d}$ and $N_{d}$ in Equation (4).

In cases of strong competition between traps ($A_{m}=A_{d}\gg A_{i}$ and $N_{d}>N_{i}$), the conclusions of Sadek and Kitis [32] can be summarized as follows:

- A strong competition from a TDDT practically removes the competition between the active traps;
- In such strong competition cases, a condition of a pseudo-superposition principle is established, causing the individual active traps to behave independently;
- The glow curve shape shows remarkable stability;
- The simulated complex TL glow curves were fitted excellently with the available analytical CGCD expressions;
- The values of the kinetic parameters evaluated with CGCD analysis were in very good agreement with the values used as input values in the simulation.

In the case of weak competition between traps ($A_{d}<A_{m},A_{i}$, $N_{d}<N_{i}$), the conclusions of these authors were:

- The weak competition from TDDT transfers the competition effects to the active traps;
- The last peak of a complex TL curve acts like an OTOR peak [33];
- The glow curve shape shows significant changes for different doses;
- However, even in these cases, the simulated complex TL glow curves fit very well with the available CGCD expressions;
- The values of the kinetic parameters evaluated with CGCD analysis were in very good agreement with the values used within the simulation;
- Only in cases where $A_{i}>A_{d},A_{m}$, the CGCD fails to produce accurate values of the activation energies.

Based on the above study, it is argued that there is no physical contradiction between using the CGCD analysis and the SP. The basic conclusion adopted in the present work is that one can use CGCD analysis on the basis of the pseudo-SP established by Sadek and Kitis [32].

## 6. Simulation of the TL Signal: Attempting to Separate Individual Peaks in the TL Glow Curve

It was concluded that it is not possible to extract mathematically the TL intensity of each peak from the numerical solution of the system of ODE (in Section 4). However, we will show that the CGCD analysis is an effective tool which can evaluate the contribution of each trap to the TL signal. We compare the mathematical and CGCD methods of analysis by the following simulation procedure:

1. Evaluate the integrated number of trapped electrons $n_{0}$ in active traps at the end of the irradiation stage;
2. Use Equation (16) to evaluate the integrated signal due to each trap during the heating stage. This is the mathematical approach, which is based on the solution of the differential equations;
3. Analyze the simulated glow curves using CGCD analysis, as an alternative method to obtain the integrated signal due to each TL peak. The results from this CGCD analysis will be compared with the results from using Equation (16).

**Figure 3:**
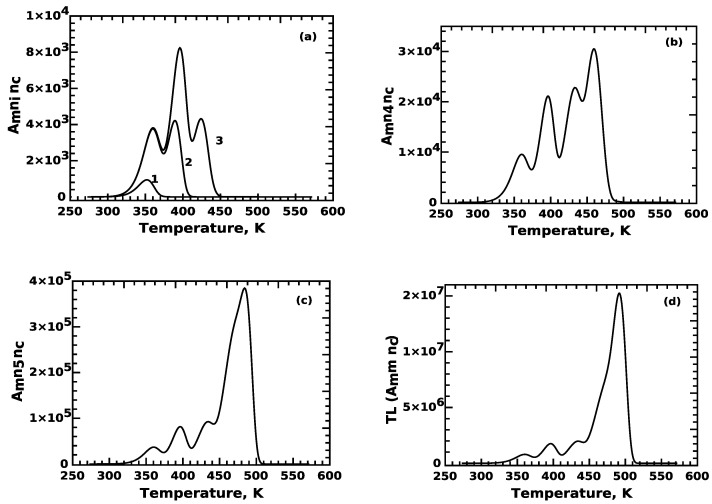
TL peak shape in the case of REFERENCE-01 glow curve, as evaluated from the numerical solutions of the model in Section 2.1. (**a**) Shapes of peaks 1, 2 and 3, (**b**) shape of peak 4, (**c**) shape of peak (5) and (**d**) total glow curve shape. It is obvious that except peak 1, all other peaks, although they must be of single-peak shape, look composite, because the electron distribution within the conduction band contributes to their numerical evaluation.

As a result of application of Equation (16), curve (1) in Figure 3a corresponds to TL peak 1, while curve (2) corresponds to TL peak 2. It is clear that peak 2 has contributions from electrons released from both $n_{1}$ and $n_{2}$.

Similarly, curve (3) of Figure 3a has contributions from traps $n_{1}$, $n_{2}$ and $n_{3}$, while curve (4) in Figure 3b has contributions from traps $n_{1}$, $n_{2}$, $n_{3}$ and $n_{4}$. Finally, peak 5 in Figure 2c has contributions from all 5 traps. The curve in Figure 3d shows the final complex TL glow curve evaluated using Equation (14).

The above results show clearly the effects of competition, namely that numerical simulations of phenomenological models with many interacting trapping levels fail to evaluate the individual TL glow peaks originating from each trapping level. In fact, it is not possible to even obtain a plot of the individual TL peaks using the ODE, and of course, it is impossible to evaluate their integrated signal.

**Figure 4:**
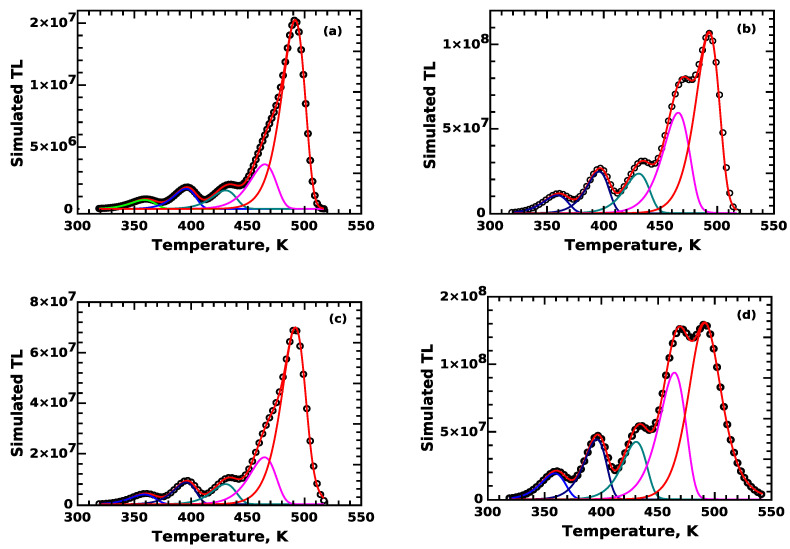
CGCD analysis results of the four REFERENCE glow curves used for simulation. (**a**) REFERENCE-01, (**b**) REFERENCE-02, (**c**) REFERENCE-03, (**d**) REFERENCE-04.

In order to complete the analysis, we use CGCD analysis to evaluate the components in the TL signal. The CGCD analysis was applied to the four reference glow curves described above, along the lines of the work by Sadek and Kitis [32]. The results are shown in Figure 4, and the results of the CGCD analysis are shown in Table 1. The three columns in Table 1 correspond to the three simulation steps 1–3 described previously in this section. The first column corresponds to the number of electron $n_{0}$ trapped in each trapping level, at the end of irradiation stage. The second column shows the integrated signal of the contribution of each term $A_{m}\;n_{i}\;n_{c}$. Finally, the third column corresponds to the integrated signal of each peak obtained from the CGCD analysis.

The first observation is that the values of the second column differ widely from the values of first and third column, while the first and third column are very close to each other. Based on the results of Table 1, it is concluded that:

(1) The numerical simulation of the ODE cannot provide an accurate measure of the integrated TL intensity for each peak;

(2) The CGCD analysis can provide a much more accurate estimate of the simulated TL intensity for each peak in a complex simulated glow curve.

## 7. Testing the Results of Simulations: Comparison of the Output and Input Parameter Values E and s

The values of the kinetic parameters *E* and *s* of each electron trap are intrinsic properties of the traps, without any dependence on the traffic of electrons in the conduction band. The big advantage of the CGCD analysis of TL signals is that it evaluates both the integrated intensity of each peak in the glow curve, as well as the kinetic parameters *E* and *s* of the individual peaks.

It is clear then that a strong test of the results from simulations is the comparison of the input values of *E* and *s* in the model, with the corresponding values obtained by the CGCD analysis.

As mentioned above, an extended initial study comparing the input and output parameters was carried out by Sadek and Kitis [32], and their conclusions were previously summarized in Section 5.

In the present work, we will expand this previous study by applying CGCD analysis to the four simulated reference glow curves (REFERENCE-01 to REFERENCE-02 described above).

The CGCD analysis is shown in Figure 4, and Table 2 shows the comparison between the input and the output values of the kinetic parameters. The output values of the activation energy *E* are the mean from all reference glow curves. The agreement between the input values of *E* and *s*, and the CGCD estimates are excellent.

The peak maximum temperatures ($T_{m}$) shown in Table 2 are remarkably stable, although the simulation parameters of the four reference glow curves differ widely. The last column contains the values of the retrapping coefficient $R=A_{n}/A_{m}$ (which corresponds to the kinetic order of the TL process). The very low *R* values for peaks 1–4 indicate first-order kinetics, which is expected due to the strong competition between traps [34].

Also of interest are the values of *R* in the case of peak 5, which increase as the competition between traps decreases. The values for reference glow curves 1 and 2 correspond to high competition between the traps, and the *R* values are very small (*R* = 0.001 and 0.01), indicating first-order kinetics [34].

In the case of reference glow curve 3, the competition is weaker, and the R value is somewhat higher (*R* = 0.05). Finally, for reference glow curve 4, the competition is the weakest, and the value of *R* = 0.82. This is in agreement with the results of Sadek and Kitis [32], who found that the last peak of the glow curve (peak 5 in our study) takes the place of the competitor, and that the last peak adopts the properties of the one trap one recombination center (OTOR) model. The value of *R* = 0.82 means that under weak competition, the TL kinetics tends to second-order kinetics.

These results establish that the stability of the *E* and *s* values during the simulation can be used as a unique criterion for simulation testing. We state this criterion as follows:

- *If the input and output values of E and s in a simulation agree with each other, the simulation is valid, and the effects of competition and of the superposition principle have been taken into account successfully;*
- *Any disagreement between input and output values does not necessary mean an invalid simulation, but it could indicate instead the appearance of new physical processes, which need additional study and interpretations.*

## 8. Testing the Results of Simulations by Using Experimental Results

A simulation without any experimental control can often lead to unrealistic results. In order to use experimental results for simulation testing, it is necessary to find experimental results which correspond to very specific and stable luminescence signals. As an example, we will consider natural quartz, which is used as a natural dosimeter in archaeological and geological dating.

According to the previous section, the first and most important test is to require the equality between the model input with the output CGCD values of the kinetic parameters of each peak. If a simulation does not reproduce the experimental parameters, then further explanation and interpretation is required. Disagreement between input and output values can provide useful information about the influence of experimental conditions on the values of the parameters.

In a simulation using a multilevel phenomenological model to study a specific material, we can further ask that the simulation reproduce well-known and generally accepted properties of the materials.

In the case of quartz, the low-temperature TL peak, known as the 110 ${}^{0}C$ peak in the literature, can act as the ideal tester of the validity of simulated results. The important general properties of this peak are listed below:

- This peak appears in all quartz samples of any origin [35];
- The activation energy of this peak in all types of quartz lies in a narrow range *E* = 0.75–0.9 eV [35];
- The kinetic order is always of the first order [35];
- The activation energy and peak maximum position $T_{m}$ remains unchanged, even after very strong external preconditioning of the sample (e.g., high-temperature annealing and the pre-dose effect [36,37,38]).

Recently, TL and OSL research groups have recognized the importance of the 110 °C peak, and its properties became the subject of inter-comparison programs between research groups (Schmidt et al. [39,40]).

**Figure 5:**
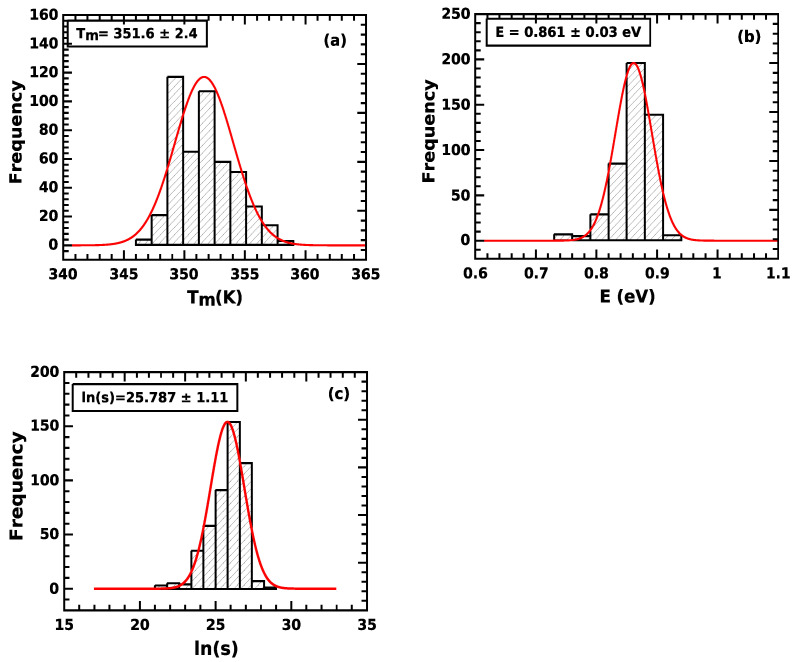
(**a**) Histogram for the position of peak maximum temperature, (**b**) histogram for the values of the activation energy and (**c**) histogram of the logarithm of the frequency factor.

In the present work, we present new experimental results with very high repeatability. The quartz sources and the experimental procedure were presented in Section 2. The measuring protocols consisted of many thermal heating cycles and temperatures, so that a large number of TL glow curves of different quartz samples was measured. The new data include many pre-conditioned aliquots, which potentially could influence the properties of the TL glow peak at 110 °C. The characteristics studied by CGCD analysis are the activation energy, peak maximum temperature, frequency factor and kinetic order. The results are shown in Figure 5. Figure 5a shows the position of peak maximum temperature T${}_{m}$ = 351.6 ± 2.4 °C, which is extremely stable considering the difference in samples and pre-conditioning of the aliquots. Figure 5b shows the value of the activation energy E = 0.861 ± 0.03 eV. This value is exactly within the limits established in the study by Pagonis et al. [35], and also agrees with the error with the corresponding values obtained in the recent inter-comparison programs ([39,40]). Finally, the values of the logarithm of frequency factor are represented in Figure 5c are also very well concentrated in a narrow region of values. This is a good achievement because the frequency factor is evaluated from the condition for the maximum, so the propagation of the errors of *E* and $T_{m}$ in the values of frequency factor has an exponential dependence. For example, an 1% error in *E* results a 25–30% error in frequency factor [1].

We consider the parameter $R=A_{n}/A_{m}$, i.e., the ratio of the retrapping over recombination coefficients. The resulting *R* values in all cases are less that 0.01, which indicates clearly first-order kinetics [14,16]. Note that the parameter *R* is more representative of the physical process than the parameter *b* used in general-order kinetics. The reason is that *R* is related to both the re-trapping and recombination coefficient, whereas the empirical parameter *b* is related to the re-trapping process only.

In conclusion, these new results show that the properties of the TL peak 110 °C are an ideal test for simulation studies of quartz. Furthermore, this type of analysis can be used as a general approach while simulating the properties of any stable dosimetric material.

The method can also be used for more complex glow curves. A well-known example is the glow curve of the most commonly used dosimeter LiF:Mg,Ti.

LiF:Mg,Ti has a glow curve which consists of five individual peaks ranging from room temperature up to 250 °C. Its glow curve remains extremely stable from the lowest possible dose up to the onset of saturation after many irradiation–readout cycles. The kinetic parameters of each glow peak, especially of peak 5, are very well known and are generally accepted by dosimetry research groups.

It is suggested that CGCD analysis of any simulation of LiF:Mg,Ti should reproduce the well-known values of its kinetic parameters. These values were extensively tested, showing an excellent inter-laboratory repeatability, in the framework of the GLOCANIN inter-comparison project [5,6].

Additionally, an extensive study of this type was conducted by Kitis et al. [41]. A substantial part of personal dosimetry services is carried out by hot gas TLD readers, under exponential heating function, which gives glow curves shapes very different than that of the linear heating. The LiF:Mg,Ti chips are used in routine monitoring within the Greek Atomic Energy Commission (GAEC). The measuring device is an automatic RADOS reader using nitrogen gas for each readout up to 573 K (300 °C) for 15 s. Prior to their readout, the chips are post-irradiation annealed at 353 K (80 °C) for 1 h. The irradiation was performed at the Secondary Standard Dosimetry Laboratory of GAEC. ${}^{137}$Cs and ${}^{60}$Co were used for the linearity tests. A batch of 10 chips was irradiated at each dose. For the quality control irradiation, a TLD irradiator with ${}^{90}$Sr/${}^{90}$Y source was used.

Using appropriate analytical TL expressions for heating under exponential function [3], Kitis et al. [41] applied the CGCD analysis to a large number (∼100) of glow curves irradiated with doses between 0.1 and 1000 mGy and also to 130 quality assurance glow curves after a dose of 5 mGy. Kitis et al. [41] showed that the glow curves shapes were the same in the whole dose region examined, Furthermore, they were able to analyze all of them using the same values of activation energy, namely 1.24 ± 0.09 eV, 1.45 ± 0.05 eV and 2.28 ± 0.02 eV, for peaks 3, 4 and 5, correspondingly. The CGCD results for the 130 quality assurance glow curves (dose 5 mGy) were 1.16 ± 0.15, 1.46 ± 0.09 and 2.16 ± 0.06 for peaks 3, 4 and 5, correspondingly. The agreement between the above values and those of the GLOCANIN project [5,6] is very good.

It is concluded that the dosimetric LiF:Mg,Ti glow curves can be used reliably for simulation testing, due to their stability and the repeatability of their kinetic parameters. However, care must be taken in two cases where substantial variation of the TL glow curve can take place. Firstly, for aliquots which are pre-conditioned by annealing for many hours between 140 and 160 °C [42,43,44], and secondly, after very high irradiation doses [45,46].

## 9. Conclusions

It is shown by numerical simulation that there is no reliable mathematical method to extract the information from simulated complex TL glow curves. It is also shown that the CGCD is the only reliable method to extract all information from simulated complex TL glow curves. Furthermore, there is no physical contradiction between the superposition principle and CGCD analysis. The CGCD analysis reproduces all characteristic of a complex glow curve very accurately, especially when the competitor removes the competition between active traps. The CGCD is also accurate for cases of low competition between the traps. The only exception is in cases when the last peak of a complex glow curve takes the role of competitor, due to absence of competition from deep traps. Furthermore, this last peak can become a peak with an OTOR behavior and second-order kinetics. The presence of strongly competing TDDT results in very stable TL glow curve shapes. As the TDDT competition weakens, the shape of the glow curves changes. The agreement between input parameter values and output values evaluated by CGCD analysis is a very powerful criterion for the validity of simulations. Experimental glow curve characteristics with high repeatability can contribute substantially to simulation testing. The most characteristic examples are the experimental properties of the 110 °C glow peak of quartz, as well as the dosimetric glow curves of LiF:Mg,Ti.

## Figures and Tables

**Table 1:** Individual peak integral evaluations. The first column corresponds to the number of electrons $n_{0}$ trapped in each trapping level, at the end of irradiation stage. The second column shows the integrated signal of the contribution of each term $A_{m}\;n_{i}\;n_{c}$ and the third column corresponds to the integrated signal of each peak obtained from the CGCD analysis.

Peak	$n_{0}$	$A_{m}\;n_{i}\;n_{c}$	CGCD Analysis
REFERENCE-01-($A_{1,2,3,4}=10^{-9}$, $A_{5}=4\times 10^{-9}$, $A_{m}=A_{d}=10^{-7}$)			
1	$2.8\times 10^{7}$	$2.60\times 10^{4}$	$2.15\times 10^{7}$
2	$4.6\times 10^{7}$	$2.02\times 10^{5}$	$4.02\times 10^{7}$
3	$4.6\times 10^{7}$	$4.13\times 10^{5}$	$4.23\times 10^{7}$
4	$1.2\times 10^{8}$	$2.17\times 10^{6}$	$1.04\times 10^{8}$
5	$2.0\times 10^{9}$	$1.94\times 10^{7}$	$4.06\times 10^{8}$
REFERENCE-02-($A_{1,2,3,4}=5\times 10^{-8}$, $A_{5}=9\times 10^{-8}$, $A_{m}=A_{d}=10^{-7}$)			
1	$4.5\times 10^{8}$	$5.02\times 10^{6}$	$3.05\times 10^{8}$
2	$9.0\times 10^{8}$	$4.00\times 10^{7}$	$6.33\times 10^{8}$
3	$9.0\times 10^{8}$	$8.72\times 10^{7}$	$6.73\times 10^{8}$
4	$2.2\times 10^{9}$	$4.83\times 10^{8}$	$1.82\times 10^{9}$
5	$3.3\times 10^{9}$	$1.42\times 10^{9}$	$2.94\times 10^{9}$
REFERENCE-03-($A_{1,2,3,4}=10^{-9}$, $A_{5}=4\times 10^{-9}$, $A_{m}=10^{-7}$, $A_{d}=10^{-10}$)			
1	$1.2\times 10^{8}$	$2.10\times 10^{6}$	$1.09\times 10^{8}$
2	$2.3\times 10^{8}$	$1.69\times 10^{7}$	$2.22\times 10^{8}$
3	$2.3\times 10^{8}$	$3.61\times 10^{7}$	$2.23\times 10^{8}$
4	$5.9\times 10^{8}$	$2.16\times 10^{8}$	$5.63\times 10^{8}$
5	$2.0\times 10^{9}$	$2.41\times 10^{9}$	$1.98\times 10^{9}$
REFERENCE-04-($A_{1,2,3,4}=5\times 10^{-8}$, $A_{5}=9\times 10^{-8}$, $A_{m}=10^{-7}$, $A_{d}=10^{-10}$)			
1	$6.6\times 10^{8}$	$1.82\times 10^{7}$	$5.40\times 10^{8}$
2	$1.3\times 10^{9}$	$1.49\times 10^{8}$	$1.13\times 10^{9}$
3	$1.3\times 10^{9}$	$3.49\times 10^{8}$	$1.19\times 10^{9}$
4	$2.3\times 10^{9}$	$2.34\times 10^{9}$	$2.95\times 10^{9}$
5	$4.3\times 10^{9}$	$7.69\times 10^{9}$	$4.97\times 10^{9}$

**Table 2:** Comparison of the kinetic parameters for each energy level in the model, as obtained using CGCD analysis. The input values of the model are compared with the output values from CGCD. The input values for E,s for each peak are common to all reference cases. The units of *E* are eV, and the units of *s* are s${}^{-1}$.

	Input Values		Output Values			
**Peaks**	**E**	**s**	**E**	**s**	$T_{m}$	**R**
1	1.0	$1.0\times 10^{13}$	1.0±0.01	$\left(1.28\pm 0.28\right)\times 10^{13}$	358.9±0.09	0.002
2	1.38	$3.9\times 10^{16}$	1.37±0.006	$\left(3.13\pm 0.64\right)\times 10^{16}$	395.8±0.20	0.001
3	1.48	$2.0\times 10^{16}$	1.48±0.013	$\left(1.92\pm 0.64\right)\times 10^{13}$	430.6±0.18	0.005
4	1.6	$2.0\times 10^{16}$	1.59±0.008	$\left(1.50\pm 0.32\right)\times 10^{13}$	464.9±0.50	0.002
5	2.01	$4.0\times 10^{19}$	2.004±0.02	$\left(3.44\pm 0.3\right)\times 10^{13}$	492.3±0.60	0.001, 0.01, 0.05, 0.82

## Data Availability

All results can be provided by the authors via E-mail.
